# The Short-Term Remission of Diabetic Nephropathy After Roux-en-Y Gastric Bypass in Chinese Patients of T2DM with Obesity

**DOI:** 10.1007/s11695-015-1666-y

**Published:** 2015-04-30

**Authors:** Hongwei Zhang, Jianzhong Di, Haoyong Yu, Xiaodong Han, Kun Li, Pin Zhang

**Affiliations:** Department of General Surgery, The Sixth People’s Hospital Affiliated to Shanghai Jiao Tong University, Shanghai, 200233 China; Department of Endocrinology and Metabolism, The Sixth People’s Hospital Affiliated to Shanghai Jiao Tong University, Shanghai, 200233 China

**Keywords:** Gastric bypass, Type 2 diabetes mellitus, Diabetic nephropathy, Microalbuminuria, Albumin/creatinine ratio

## Abstract

The study aimed to examine the effect of gastric bypass on diabetic nephropathy (DN) in Chinese patients. A retrospective study of 101 samples with DN stages I to IV underwent Roux-en-Y gastric bypass. Anthropometric and biochemical parameters were assessed during a 1-year follow-up. Binary logistic regression analysis was used for predictors and ROC for cutoff points. There was significant reduction of medication for diabetes and hypertension. Overall remission of T2DM was 80.2 %. Preoperative albumin/creatinine ratio (ACR) and serum creatinine (SCr) levels could be predictors for DN remission, and the cutoff points were 126 mg/g cr and 57 μmol/L, respectively. Gastric bypass showed improvement of T2DM and DN. Gastric bypass may be more beneficial for those with lower preoperative ACR and SCr.

**Electronic supplementary material:**

The online version of this article (doi:10.1007/s11695-015-1666-y) contains supplementary material, which is available to authorized users.

The incidence of diabetes mellitus has risen rapidly in the past few decades, and its prevalence has increased from less than 1 % in the Chinese population in the 1980s [1] to 11.6 % in the Chinese adult population [2]. Even more serious is that only 25.8 % of these patients received treatment for diabetes, and only 39.7 % of those treated had adequate glycemic control. These findings indicate the importance of diabetes as a public health problem in China with the potential for a major epidemic of diabetes-related complications.

Diabetic nephropathy (DN), also known as diabetic glomerulosclerosis or diabetic kidney disease, is one of the major microvascular complications of type 2 diabetes mellitus (T2DM) [2–4], and is a leading cause of end-stage renal disease (ESRD) with high mortality [5, 6]. Current strategies for the treatment of DN to delay progression involve control of blood glucose and metabolic abnormality [7].

Metabolic surgery has been approved as an effective and potentially useful treatment for T2DM with obesity, especially the Roux-en-Y gastric bypass (RYGB) [8–10]. Several observational studies have shown impressive improvements in glycemic as well as in metabolic disorder, and diabetic complications have shown to be partially reversible due to weight loss and diabetes control [11, 12]. Our prior studies have also shown significant improvement in T2DM including erectile dysfunction and obstructive sleep apnea [13–15].

Though the evidence suggests that RYGB might be a potential treatment for DN, it remains unclear whether the surgery improves progression or provides remission for incipient and overt nephropathy. Therefore, the aim of our study was to investigate whether the RYGB is appropriate for DN.

## Methods

A retrospective study was made between February 2011 and December 2013. A total of 157 patients had RYGB during this period, and 101 T2DM patients with different DN stages were enrolled. The inclusion criteria included (1) age from 18 to 65 years, (2) BMI over 28 kg/m^2^, (3) a diagnosis of T2DM based on the criteria of the American Diabetes Association (ADA), and (4) a fasting C-peptide by the oral glucose tolerance test >1 ng/mL and a ratio of peak to fasting value >2 ng/mL. Those with established diagnoses of type 1 diabetes, latent autoimmune diabetes in adults, malignancy, debilitating disease, unresolved psychiatric illness, or substance abuse were excluded from the study.

DN is divided into five stages according to the Morgenson standard [16]: (I) hyperfunction with raised glomerular filtration rate (GFR) and increased kidney volume, (II) normoalbuminuria with the thickening of the glomerular basement membrane (GBM) and expansion of the mesangial matrix, (III) incipient DN with microalbuminuria (MAU) 30–299 mg/day, (IV) overt DN with MAU >300 mg/day, and (V) uremia with ESRD. Patients with DN stage V were excluded from this study for surgical contraindication. Remission in DN stages III and IV was defined as MAU <30 mg/day. This study focused on patients in DN stage III and IV, which are more likely to develop end-stage renal disease.

Medical history, current medications, anthropometric evaluations including weight, BMI, percent of excess weight loss (%EWL), waistline, and hipline were recorded before and after bypass surgery. Biochemical parameters were evaluated by obtaining blood samples after an overnight fast and included fasting glucose, postprandial glucose followed by OGTT, fasting C-peptide, glycated hemoglobin (HbA1c), glycosylated serum protein (GSP), lipid profile (cholesterol, triglyceride, high-density, and low-density lipoprotein), and renal function profile (blood urea nitrogen [BUN], serum creatinine [SCr], and uric acid [UA]). In addition, the screening of DN with GFR, urine albumin/creatinine ratio (ACR), and 24-h microalbuminuria were evaluated. Insulin resistance level and β-cell function were measured by the homeostasis model assessment insulin resistance (HOMA-IR) using the formula mIU/mmol/L2 = fasting insulin (mIU/L) × fasting glucose (mmol/L) / 22.5 and the homeostasis model assessment β-cell (HOMA-β) using % = 20 × fasting insulin (mIU/L) / fasting glucose (mmol/L) − 3.5) (%).

Remission of T2DM was defined as an HbA1c level <6.5 % and a fasting glucose concentration <7.0 mmol/L for 1 year or more without active pharmacologic intervention [17]. Plasma glucose concentrations were measured using the glucose oxidase method. Serum insulin and C-peptide levels were quantified using radioimmunoassays with specific insulin and C-peptide detection kits according to the manufacturer’s instructions (Beijing North Institute of Biological Technology, Beijing, China). HbA1c was measured by HPLC (Menarini, former reference range 4.0–6.0 %). GSP was measured by ELISA (Hitachi 7100, Tokyo, Japan). The serum lipid and renal function profile were measured using standard commercial methods on a parallel, multichannel analyzer (Hitachi 7600–020, Tokyo, Japan). ACR and 24-h MAU were measured by chemiluminescence immunoassay (Siemens, DCA Vantage, Germany), and GFR was measured by 99 m Tc-DTPA (Siemens, SPECT, E CAM Single, Germany).

Of the 101 patients, 45 (44.6 %) were female. The mean age of all participants was 47.6 ± 11.9 years, and the mean duration of T2DM was 7.63 ± 4.84 years. Hypertension was diagnosed in 40 (39.6 %). All patients were divided into four groups according to their DN stage diagnosis.

Nearly 50 % were treated with insulin and more than 60 % with oral diabetes medications in each group preoperatively. Patients in DN stage I with hypertension (25.8 %) had the least proportion of antihypertensives (12.9 %). Most of the antihypertensives taken were angiotensin-converting enzyme inhibitor (ACEI) or/with angiotensin receptor blocker (ARB). Antihyperlipidemia medication was taken by 61.9 and 60 % separately in DN stages III and IV while in DN stages I and II, it was 48.4 and 35.3 %, respectively (Table 1).

**Table 1:** Characteristics of the participants

	DN1	DN2	DN3	DN4
Gender (F/M)	16:15	08:26	11:10	10:05
Age (years)	41.35 ± 9.37	54.91 ± 10.23	47.62 ± 13.70	44.13 ± 8.70
Duration (years)	4.87 ± 2.85	9.94 ± 4.57	7.18 ± 5.60	8.33 ± 4.92
Hypertension	8 (25.8 %)	14 (41.2 %)	11 (52.4 %)	7 (46.7 %)
Medication use before surgery				
Insulin	10 (47.6 %)	17 (50.0 %)	9 (42.9 %)	13 (46.7 %)
Oral diabetes medications	19 (61.3 %)	25 (73.5 %)	13 (61.9 %)	9 (60.0 %)
Antihypertensives	4 (12.9 %)	12 (35.3 %)	9 (42.9 %)	6 (40.0 %)
Antihyperlipidemia	15 (48.4 %)	12 (35.3 %)	13 (61.9 %)	8 (53.3 %)
Medication use 1 year after surgery				
Insulin	1 (3.2 %)	2 (5.9 %)	1 (4.8 %)	1 (6.7 %)
Oral diabetes medications	2 (6.5 %)	2 (5.9 %)	2 (9.5 %)	1 (6.7 %)
Antihypertensives	2 (6.5 %)	5 (14.7 %)	2 (9.5 %)	1 (6.7 %)
Antihyperlipidemia	1 (3.2 %)	1 (2.9 %)	1 (4.8 %)	0

### Surgical Technique

All patients had laparoscopic Roux-en-Y gastric bypass (LRYGB) by the same surgical team with over 50 cases of experience. The patient was placed in reverse Trendelenburg position and the surgeon positioned between the patient’s legs. Five trocars are inserted under direct laparoscopic vision. A gastric pouch of approximately 30 mL is created with mechanical sutures preventing the transection of the left gastric artery. The angle of Treitz is then identified, and a 100-cm-long jejunal loop (biliopancreatic limb) is ascended anterior to the colon and anastomosed to the gastric pouch with mechanical linear suture (30 mm). A lateral jejuno-jejunal anastomosis adjacent to gastro-jejunal anastomosis is made 100 cm away from the previous nastomosis (alimentary limb) with a mechanical linear stapler.

### Statistics Analysis

All statistics were calculated with SPSS statistical software (version 20.0; SPSS Inc., Armonk, NY, USA). Paired *t* tests were used to compare variables before and after the study. Contingency tables of categorical variables were analyzed by Fisher’s exact test (grade and gender in remission of DN stages III and IV). Correlations between clinical parameters and MAU were analyzed by partial correlation analysis. Multiple stepwise logistic regression analysis was performed to assess the independent predictive effects of the variables on remission of DN. The measure index was set as blood pressure, albumin/creatinine ratio and serum creatinine, and the remission standard as MAU <30 mg/day. The predictors’ cutoff point was evaluated by Youden’s Index with the receiver operating characteristic (ROC) curve. Data are presented as the mean ± SD. Statistical significance was defined as *P* < 0.05.

## Results

Four groups are comparable in BMI (*F* = 1.529, *P* = 0.212), patients in DN stage II were older (*F* = 0.915, *P* = 0.000) and longer T2DM duration (*F* = 6.975, *P* = 0.000). One year after surgery, there was a significant reduction of each type of medication used in each group (Table 1).

The overall remission of T2DM was 80.20 %, and in DN stage I, it was 95.2 %, DN stage II was 86.7 %, DN stage III was 23.8 %, and DN stage IV was 46.7 %. Changes in anthropometric and biochemical parameters of all patients before and after surgery are shown in Table 2.

**Table 2:** Changes in variables of patients before and 1 year after gastric bypass

Variables	Baseline				1 year after surgery			
	DN1	DN2	DN3	DN4	DN1	DN2	DN3	DN4
Weight (kg)	85.85 ± 11.92	80.09 ± 10.45	89.05 ± 11.57	90.00 ± 13.31	67.83 ± 9.47**	61.31 ± 7.34**	70.03 ± 9.46**	69.08 ± 11.97**
BMI (kg/m^2^)	30.54 ± 3.05	30.16 ± 2.89	31.74 ± 3.86	31.72 ± 3.20	24.26 ± 2.17**	23.43 ± 2.27**	24.74 ± 2.47**	24.47 ± 2.38**
%EWL	–	–	–	–	134.9 ± 57.1**	162.96 ± 108.13**	135.74 ± 105.40*	126.58 ± 74.94*
MAU (mg/24 h)	15.95 ± 11.37	13.26 ± 7.89	83.73 ± 59.13	1052.32 ± 954.77	9.97 ± 4.48	8.46 ± 4.00**	17.94 ± 14.73**	513.46 ± 730.36
GFR (mL/min)	106.47 ± 25.87	98.69 ± 15.97	110.81 ± 21.23	91.55 ± 31.39	93.45 ± 22.74	89.36 ± 26.52	108.49 ± 29.77	95.98 ± 30.16
ACR (mg/ g cr)	12.03 ± 13.70	10.84 ± 7.39	75.13 ± 51.40	713.82 ± 794.03	4.50 ± 5.38**	6.54 ± 4.75**	15.11 ± 11.87**	376.50 ± 613.71
SBP (mmHg)	128.65 ± 10.26	131.35 ± 17.05	137.90 ± 15.07	134.53 ± 10.65	119.04 ± 15.88**	122.00 ± 13.22*	120.53 ± 8.01**	121.67 ± 8.34**
DBP (mmHg)	83.03 ± 8.01	80.79 ± 11.40	122.14 ± 160.91	81.20 ± 7.06	75.42 ± 9.69**	75.92 ± 9.35*	77.67 ± 6.78	73.08 ± 9.39*
BUN (mmol/L)	4.45 ± 1.07	5.22 ± 1.59	4.84 ± 1.46	5.71 ± 2.03	4.58 ± 1.33	5.63 ± 1.38	5.21 ± 1.33	5.69 ± 2.44
SCr (umol/L)	62.13 ± 17.46	62.35 ± 16.44	58.90 ± 16.78	79.13 ± 29.51	61.58 ± 12.29	55.60 ± 9.22	59.00 ± 19.12	68.75 ± 30.14
UA (umol/L)	352.06 ± 98.03	338.94 ± 85.84	377.00 ± 109.74	382.40 ± 132.84	310.67 ± 95.03	285.56 ± 73.91*	329.80 ± 72.32	327.25 ± 112.89
FBG (mmol/L)	9.03 ± 3.13	8.52 ± 2.56	8.66 ± 1.69	8.28 ± 2.69	5.73 ± 1.11**	6.16 ± 1.28**	5.63 ± 1.02**	6.37 ± 1.62*
PBG (mmol/L)	13.66 ± 4.62	12.90 ± 4.31	13.81 ± 3.39	12.96 ± 5.16	7.56 ± 2.63**	7.50 ± 3.57**	7.50 ± 2.35**	8.00 ± 3.65**
HbA1c (%)	8.33 ± 2.05	8.70 ± 2.19	8.41 ± 1.85	7.84 ± 1.72	5.98 ± 1.14**	6.33 ± 0.86**	5.92 ± 0.59**	6.04 ± 0.81**
HOMA-IR	8.85 ± 3.43	12.15 ± 22.26	9.39 ± 7.85	23.95 ± 46.08	1.69 ± 1.05**	2.53 ± 3.59*	1.76 ± 0.87**	5.37 ± 11.46**
HOMA-β (%)	124.21 ± 122.47	143.62 ± 231.45	112.32 ± 112.78	174.31 ± 244.76	71.60 ± 45.42*	72.71 ± 62.69	80.73 ± 59.45	172.63 ± 386.17
CH (mmol/L)	4.78 ± 1.12	4.97 ± 0.89	5.10 ± 1.11	4.91 ± 1.02	4.00 ± 0.75**	4.07 ± 0.94**	3.87 ± 0.84**	4.14 ± 0.72*
TG (mmol/L)	2.89 ± 3.66	2.27 ± 1.93	2.95 ± 2.25	2.56 ± 1.40	1.03 ± 0.41**	0.92 ± 0.33**	1.14 ± 0.39**	0.98 ± 0.26**
HDL (mmol/L)	0.92 ± 0.20	1.09 ± 0.26	1.01 ± 0.23	0.98 ± 0.18	1.15 ± 0.28**	1.31 ± 0.32**	1.20 ± 0.24*	1.38 ± 0.27**
LDL (mmol/L)	2.80 ± 0.98	2.85 ± 0.84	2.85 ± 0.92	2.82 ± 0.85	2.43 ± 0.59	2.20 ± 0.72**	2.21 ± 0.56*	2.10 ± 0.46*

Data represent means ± SD or median (interquartile range) or percentages

*BMI* body mass index, *%EWL* percentage of excess weight loss, *MAU* microalbuminuria, *GFR* glomerular filtration rate, *ACR* albumin/creatinine ratio, *HR* heart rate, *SBP* systolic blood pressure, *DBP* diastolic blood pressure, *BUN* blood urea nitrogen, *SCr* serum creatinine, *UA* uric acid, *FPG* fasting plasma glucose, *PBG* postprandial glucose, *HbA1c* hemoglobin A1c, *HOMA-IR* homeostasis model assessment-insulin resistance, *HOMA-β* homeostasis model assessment β-cell, *CH*: Cholesterol, *TG* triglycerides, *HDL* high-density lipoprotein, *LDL* low-density lipoprotein

*P < 0.05; **P < 0.01

The overall remission of DN stages III and IV was 58.3 %. There was no significant difference in age or BMI between two groups. Analysis showed that the remission parameter microalbuminuria was correlated with albumin/creatinine ratio, systolic blood pressure (SBP), serum creatinine, Homa-IR, Homa-β, fasting C-peptide, and cholesterol levels (Table 3). According to the DN remission standard for incipient (stage III) and overt (stage IV) nephropathy, the preoperative clinical variables related to diabetes were assessed for their predictive values (Table 4). Analysis showed that in DN patients with stage III, there were lower microalbuminuria, albumin/creatinine ratio, systolic blood pressure, blood urea nitrogen, and serum creatinine levels compared with those without remission. Preoperative BMI, diabetes duration, waistline, and GFR had no significant differences between the two groups. The results of multivariate regression analysis confirmed that systolic blood pressure, serum creatinine and albumin/creatinine ratio were clinical predictors for DN remission (Table 5). Further analysis for predicting the DN remission using ROC showed that AUC in serum creatinine and albumin/creatinine ratio was 0.770 and 0.917 with the cutoff point of 57 μmol/L in serum creatinine and 126 (mg/g cr) in albumin/creatinine ratio (Table 6).

**Table 3:** The correlation test for MAU in patients with DN III/IV

	MAU	
	*r*	*P*
Age (years)	−0.063	0.543
Duration (years)	0.099	0.346
GFR (mL/min)	−0.161	0.062
ACR (mg/g cr)	0.962	0.000**
Weight (kg)	0.040	0.479
BMI (kg/m^2^)	0.105	0.065
%EWL	−0.003	0.961
Waistline (cm)	0.041	0.474
Hipline (cm)	0.015	0.794
HR (bpm)	0.069	0.229
SBP (mmHg)	0.126	0.026*
DBP (mmHg)	−0.005	0.936
BUN (mmol/L)	0.110	0.053
SCr (μmol/L)	0.256	0.000**
UA (μmol/L)	0.067	0.242
FBG (mmol/L)	0.072	0.207
PBG (mmol/L)	0.058	0.310
HbA1c (%)	0.049	0.310
GSP (%)	−0.069	0.226
HOMA-IR	0.336	0.000**
HOMA-β (%)	0.264	0.000**
CP 0 (ng/mL)	0.112	0.049*
CH (mmol/L)	0.173	0.002**
TG (mmol/L)	0.051	0.370
HDL (mmol/L)	0.083	0.143
LDL (mmol/L)	0.083	0.143

*BMI* body mass index, *%EWL* percentage of excess weight loss, *MAU* microalbuminuria, *GFR* glomerular filtration rate, *ACR* albumin/creatinine ratio, *HR* heart rate, *SBP* systolic blood pressure, *DBP* diastolic blood pressure, *BUN* blood urea nitrogen, *SCr* serum creatinine, *UA* uric acid, *FPG* fasting plasma glucose, *PBG* postprandial glucose, *HbA1c* hemoglobin A1c, *GSP* glycosylated serum protein, *CP 0* fasting C-peptide, *HOMA-IR* homeostasis model assessment-insulin resistance, *HOMA-β* homeostasis model assessment β-cell, *TG* triglycerides, *CH* cholesterol, *HDL* high-density lipoprotein, *LDL* low-density lipoprotein

**Table 4:** Comparison of preoperative factors of DN remission in grade III and IV

Factor	Remission	No remission	*P* value
DN grade (III/IV)	19:2	2:13	0.000**
Gender (F/M)	11:10	4:11	0.123
Age (years)	46.48 ± 13.60	45.73 ± 9.31	0.856
Duration (years)	6.85 ± 5.58	8.80 ± 4.78	0.280
Weight (kg)	88.79 ± 11.72	90.37 ± 13.09	0.706
BMI (kg/m^2^)	31.94 ± 3.86	31.44 ± 3.17	0.682
Waistline (cm)	106.14 ± 9.96	104.53 ± 8.69	0.618
Hipline (cm)	109.05 ± 7.43	107.00 ± 6.29	0.392
MAU (mg/24 h)	132.32 ± 201.92	984.30 ± 995.50	0.005**
GFR (mL/min)	109.89 ± 24.56	92.78 ± 28.73	0.067
ACR (mg/g cr)	120.37 ± 169.61	650.48 ± 820.04	0.026*
HR (bpm)	77.00 ± 6.62	79.27 ± 3.41	0.234
SBP (mmHg)	129.28 ± 15.35	138 ± 9.82	0.049*
DBP (mmHg)	122.14 ± 160.91	81.20 ± 7.06	0.334
BUN (mmol/L)	4.72 ± 1.45	5.87 ± 1.96	0.049*
SCr (μmol/L)	57.62 ± 16.11	80.93 ± 28.61	0.010**
UA (μmol/L)	372.00 ± 110.74	389.40 ± 131.00	0.669
FBG (mmol/L)	8.67 ± 1.67	8.27 ± 2.70	0.585
PBG (mmol/L)	13.78 ± 3.46	13.00 ± 5.11	0.586
CP 0 (ng/mL)	2.73 ± 1.25	3.04 ± 1.86	0.550
HbA1c (%)	8.27 ± 1.83	8.05 ± 1.79	0.722
GSP (%)	19.45 ± 4.86	17.97 ± 4.00	0.353
HOMA-IR	8.81 ± 7.30	24.76 ± 45.92	0.125
HOMA-β (%)	105.93 ± 108.72	1042.83 ± 3335.05	0.204
CH (mmol/L)	5.11 ± 1.13	4.90 ± 0.99	0.574
TG (mmol/L)	2.85 ± 2.28	2.71 ± 1.36	0.842
HDL (mmol/L)	1.02 ± 0.23	0.95 ± 0.20	0.339
LDL (mmol/L)	2.85 ± 0.92	2.82 ± 0.85	0.923

*BMI* body mass index, *MAU* microalbuminuria, *GFR* glomerular filtration rate, *ACR* albumin/creatinine ratio, *HR* heart rate, *SBP* systolic blood pressure, *DBP* diastolic blood pressure, *BUN* blood urea nitrogen, *SCr* serum creatinine, *UA* uric acid, *FPG* fasting plasma glucose, *PBG* postprandial glucose, *HbA1c* hemoglobin A1c, *GSP* glycosylated serum protein, *CP 0* fasting C-peptide, *HOMA-IR* homeostasis model assessment-insulin resistance, *HOMA-β* homeostasis model assessment β-cell, *TG* triglycerides, *CH* cholesterol, *HDL* high-density lipoprotein, *LDL* low-density lipoprotein

Data represent means ± SD or median (interquartile range) or percentages

*P < 0.05; **P < 0.01

**Table 5:** Multiple logistic regression analysis of variables at baseline

	*B*	SE	*P* value	Exp(B)
SBP	−0.208	0.087	0.017	0.812
SCr	0.093	0.044	0.034	1.098
ACR	0.005	0.002	0.019	1.005

Predicted Probability is of membership for none remission

*B* coefficient of regression, *SE* standard error, Exp(B) odd ration, *SBP* systolic blood pressure, *SCr* serum creatinine, *ACR* albumin/creatinine ratio

**Table 6:** ROC for remission of DN in predictors

	AUC	*p* valve	95 % CI		Cutoff point
SBP	0.4	0.312	0.212	0.588	–
SCr	0.77	0.006	0.616	0.923	57
ACR	0.917	0.000	0.826	1.000	126

SBP had no predictive value (AUC < 0.5). For SCr (AUC = 0.77) and ACR (AUC = 0.917), ACR had best predictive value and accuracy for predict DN remission, AUC >0.9

*SBP* systolic blood pressure, *SCr* serum creatinine, *ACR* albumin/creatinine ratio, *AUC* area under curve

## Discussion

Recently, there have been many encouraging reports suggesting that gastric bypass is an effective, stand-alone procedure for T2DM with obesity. However, before discussing DN remission, the primary concern should be about safety. In our study, all surgeries were performed by one team with experience of over 50 cases of LRYGB. An important aspect in the learning curve for successful metabolic surgery, according to Huang [18], is having experience of approximately 30 cases. Common complications such as fistula, obstruction, stenosis, and hemorrhage did not occur in our study. In addition, there was no acute renal failure in the patients with DN stage III or IV.

The primary aim of DN treatment is currently focused on delaying its progression by improving the control of T2DM and of hemodynamic abnormalities [8]. Evidence shows that DN is characterized by structural and functional changes, which show an imbalance in afferent and efferent arteriolar resistance and may result in increased glomerular hydrostatic pressure and hyperfiltration [19, 20].

The present study advocates that vascular endothelial dysfunction leads to changes in the renal vascular system and contributes to the development of DN. Good glycemic control is effective in reducing diabetic microvascular complication [21]. Hemodynamic dysfunction could be another factor resulting in DN, and diabetic endothelial nitric oxide synthase knockout mice develop more severe glomerular lesions and proteinuria compared to wild-type mice [22].

A recently described mechanism of proximal tubule injury that involved the formation of advanced glycation end-products (AGE) by nonenzymatic binding of glucose to proteins, lipids, and nucleic acids can lead to alteration of protein structure and function, oxidative stress, and expression of proinflammatory cytokines and growth factors [23], which cause the development of DN [24]. In addition, evidence suggests that genetic predisposition, hyperglycemia, hypertension, and dyslipidemia, all factors associated to metabolic syndrome, contribute to the induction and progression of DN [25]. Thus, controlling and improving metabolic syndrome could be the key link to DN treatment. However, current strategies including diabetic medicines, antihypertensives, and antilipid medicine are not effective.

This study confirms the remarkable efficacy of gastric bypass for treatment of T2DM with obesity. The percentage of female patients participating was 44.6 %, which is lower than in other studies. The overall remission of T2DM after 1 year was 80.20 %. The central obesity status, hypertension, and lipid levels were significantly improved in all DN groups. Further analysis showed that patients in DN stage I were younger with shorter diabetes duration, less insulin resistance, and better blood pressure control compared with those in DN stages III and IV before surgery. Despite the differences in the four groups, the 1-year follow-up showed that gastric bypass can also lead to similar recovery of metabolic disorder. Although HOMA-IR and HOMA-β in DN stage IV showed impressive improvement, they were significantly higher than in the other three groups 1 year following surgery, which represents greater insulin resistance and poor β-cell function indicating progression of DN. Furthermore, the medication used for diabetes, hypertension, and hyperlipidemia was reduced significantly in all groups after surgery.

Most patients with hypertension in our study used ACEI and/or ARB, which could reduce the risk of albuminuria to control blood pressure [26]. After surgery, antihypertensive medication was significantly reduced and most patients became well controlled with blood pressure under 130/80 mmHg. A study by Pohl et al. [27] indicated that a target systolic blood pressure of 120–130 mmHg provided tremendous benefit to renal outcomes. Our study found that patients in DN stages I to IV maintained continuous improvement for metabolic disorder in 1-year short term. In addition, weight loss and better diabetic control improved the lifestyle of patients and also provided further benefit by slowing down the progression of DN.

The renal function index including serum creatinine, blood urea nitrogen, uric acid, and GFR had no significant changes. However, microalbuminuria and albumin/creatinine ratio were significantly reduced in DN stages I, II, and III, but not in patients in stage IV. The mean level reduced continuously. The microalbuminuria reflects the glomerular filtration function due to the change in endothelial cell permeability and glomerular basement membrane. The GFR is affected by several factors including the glomerular filtration function, glomerular hydrostatic pressure, and colloid osmotic pressure. Therefore, we speculate that gastric bypass could improve the capillary membrane permeability, but the complex connection between capillary hemodynamics and gastric bypass still needs further research.

Patients with DN at the time of T2DM diagnosis can go unrecognized for years. Albuminuria is common among patients with established diabetes, and becomes more prevalent with worsening glucose tolerance [28]. DN stages III and IV, which are also called incipient and overt nephropathy, are more likely to develop end-stage renal disease [29]. Therefore, our study has focused on the change of microalbuminuria especially in DN stages III and IV following surgery. The remission of DN (decrease in MAU to <30 mg/24 h) could slow the progression of nephropathy and may prevent the development of end-stage renal disease. As for patients in stages I and II, DN were controlled by reducing of GFR and microalbuminuria 1 year after surgery.

Our study found that patients in DN stage III are more likely to get remission compared with stage IV, but gender, age, and diabetic duration were not correlated. The renal function index including microalbuminuria, albumin/creatinine ratio, serum creatinine, and blood urea nitrogen had a significant difference in both patients with and without remission before surgery. Further analysis shows significant correlation with albumin/creatinine ratio, serum creatinine, systolic blood pressure, cholesterol and insulin sensitivity, and insulin resistance. This result confirms the relationship between DN and metabolic disorder.

In our study, patients with increased baseline of systolic blood pressure, serum creatinine, or albumin/creatinine ratio are less likely to achieve remission of DN, and the ROC curve revealed that serum creatinine and albumin/creatinine ratio are potential predicators for remission failure of incipient and overt DN. Youden’s Index showed that the cutoff point for serum creatinine and albumin/creatinine ratio were 57 μmol/L and 126 mg/g cr, respectively. As a normal value of the serum creatinine cutoff point with less applicability, the albumin/creatinine ratio cutoff point may have greater clinical value. Although most patients in stage IV did not reach the remission standard, the reduction of microalbuminuria helped to stabilize DN.

Our study has several limitations such as retrospective design, small population size, and the limited follow-up duration which may have interfered with the results. The study by Alexander et al. showed that loss of excess body weight had the most positive effect in patients with obesity-related focal segmental glomerulosclerosis [30], which differs from our study. Therefore, a study with a long-term follow-up and a large population sample is necessary to strengthen and establish the cutoff values for serum creatinine and albumin/creatinine ratio. In addition, a further study with prospective design is required to confirm whether DN remission after RYGB is a temporary result or has a long-term effect. It is also necessary to perform a pathology and pathophysiology study of DN before and after surgery.

In conclusion, the beneficial results observed in our study demonstrate that gastric bypass should be considered in Chinese patients with T2DM and obesity. The RYGB shows the possibility of slowing down progression of diabetic nephropathy. Furthermore, albumin/creatinine ratio could help predict the remission of DN.

## Electronic supplementary material

ESM 1 (GIF 136 kb)

High resolution image (TIFF 163 kb)
