# Supplementary figures and images for: The Short-Term Remission of Diabetic Nephropathy After Roux-en-Y Gastric Bypass in Chinese Patients of T2DM with Obesity
Source: Obes Surg. 2015 Apr 30;25(7):1263–70. doi: 10.1007/s11695-015-1666-y (PMC4460269; doi:10.1007/s11695-015-1666-y)

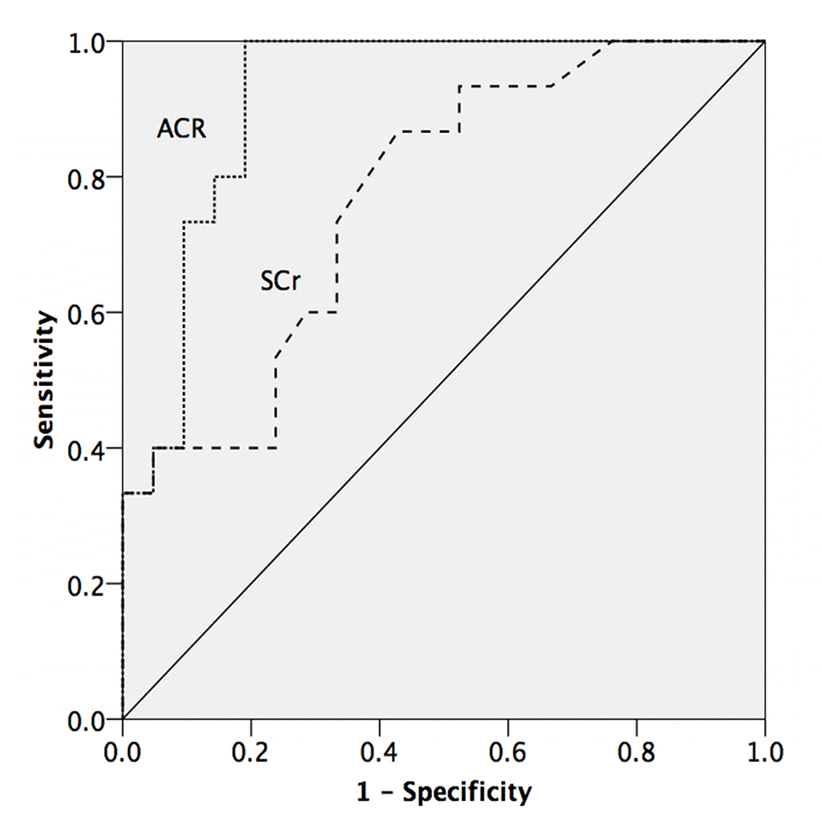

Supplement: Supplementary file 1 — (GIF 136 kb) [file 11695_2015_1666_Fig1_ESM.gif]

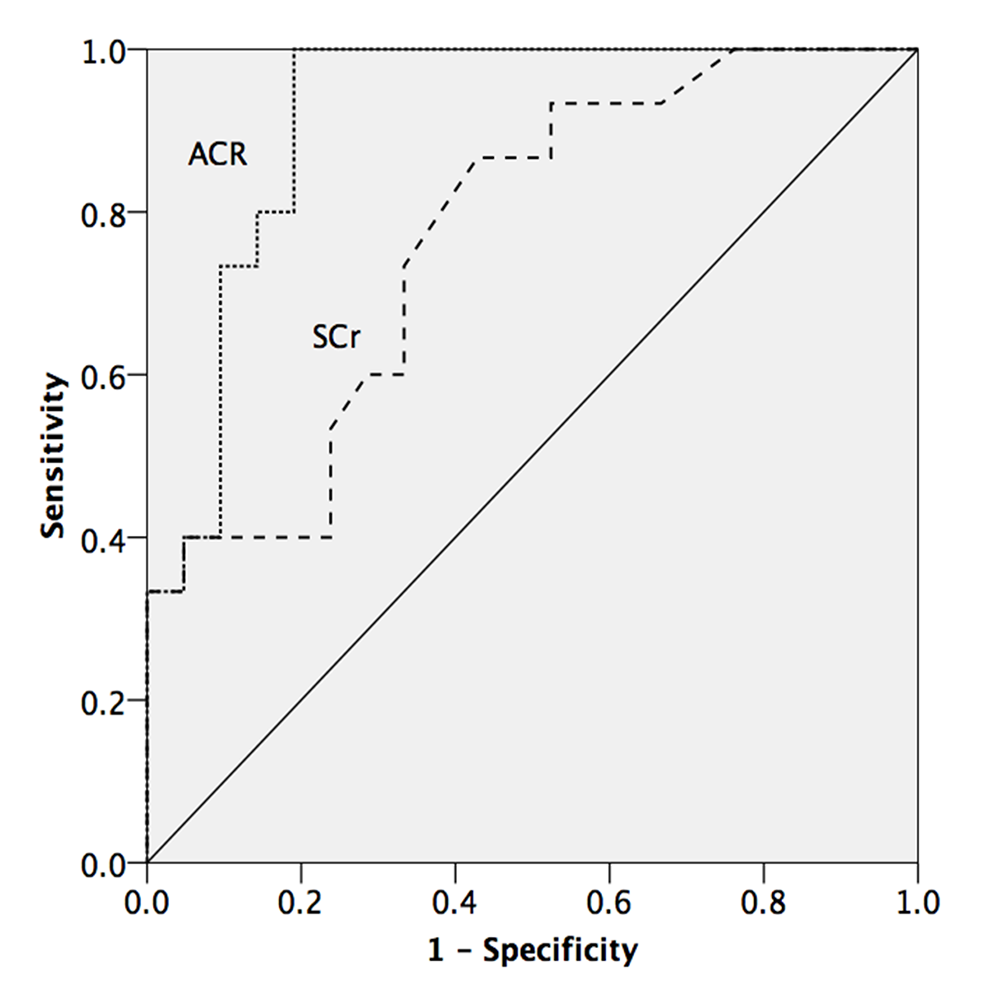

Supplement: Supplementary file 2 — High resolution image (TIFF 163 kb) [file 11695_2015_1666_MOESM1_ESM.tif]
